# On Recent Methods of Treating Diseased Pulp

**Published:** 1899-05

**Authors:** 

**Affiliations:** Professor of Dentistry in the German University, Prague


### On Recent Methods of Treating Diseased Pulp.

BY DR. BŒNNECKEN, PROFESSOR OF DENTISTRY IN THE GERMAN UNIVERSITY, PRAGUE.

Specially translated for the British Dental Journal.

A year ago I gave the results of my own observation in 500 cases of diseased pulp treated by two new methods: 1. Munification of the pulp with formaldehyde after cauterisation with arsenic; 2. Callahan's sulphuric acid treatment of gangrenous pulp. During the past year I have treated all cases (about 400) of acute and chronic pulpitis in molar and præmolar teeth in the manner detailed below. Of course, all cases of purulent destruction of the pulp or where gangrene had set in were rigidly excluded from the treatment.

In such cases the canals most be cleared and sterilized and then treated by the sulphuric acid process as described later on.

In the "formol" treatment, as it is called for brevity, the pulp cavity must be well washed out with 5 or 10 percent formalin and then filled with formal paste, which must then be carefully pressed down with a plug of wool. If the paste be fairly consistent, a cap of phosphate may be added and the permanent filling proceeded with at once. Success depends to a great extent on the purity of the formalin and the consistency of the paste. For the latter Scheurer's preparation, which is put up in tubes, is not only the best, but also the most easily manipulated. Although it contains no cocaine, the oil of cloves in it acts as an anodyne. Formaldehyde must never be applied direct to an exposed pulp, i. e., without previously applying arsenic. Indeed, it is not going too far to say that arsenic is and remains the sovereign treatments for inflamed pulp; and the only subsequent treatment which is absolutely free from objection is the total removal of the pulp followed by antiseptic filling of the roots.

Callahan's treatment of gangrenous pulp consists in the application of 5 percent sulphuric acid, with sodium peroxide as neutraliser. The canals are then filled with formol. In seven to fourteen days' time the process should be repeated, followed by cement capping and permanent filling. The results are excellent.—*Vierteljahrsschft f. Zahnhlkde.*

